# Phospholipid-Derived Fatty Acids and Quinones as Markers for Bacterial Biomass and Community Structure in Marine Sediments

**DOI:** 10.1371/journal.pone.0096219

**Published:** 2014-04-25

**Authors:** Tadao Kunihiro, Bart Veuger, Diana Vasquez-Cardenas, Lara Pozzato, Marie Le Guitton, Kazuyoshi Moriya, Michinobu Kuwae, Koji Omori, Henricus T. S. Boschker, Dick van Oevelen

**Affiliations:** 1 Department of Ecosystem Studies, Royal Netherlands Institute of Sea Research (NIOZ), Yerseke, The Netherlands; 2 Department of Marine Microbiology, Royal Netherlands Institute of Sea Research (NIOZ), Yerseke, The Netherlands; 3 School of Natural Science & Technology, Kanazawa University, Kakuma-machi, Kanazawa, Japan; 4 Center for Marine Environmental Studies (CMES), Ehime University, Matsuyama, Ehime, Japan; Scottish Association for Marine Science, United Kingdom

## Abstract

Phospholipid-derived fatty acids (PLFA) and respiratory quinones (RQ) are microbial compounds that have been utilized as biomarkers to quantify bacterial biomass and to characterize microbial community structure in sediments, waters, and soils. While PLFAs have been widely used as quantitative bacterial biomarkers in marine sediments, applications of quinone analysis in marine sediments are very limited. In this study, we investigated the relation between both groups of bacterial biomarkers in a broad range of marine sediments from the intertidal zone to the deep sea. We found a good log-log correlation between concentrations of bacterial PLFA and RQ over several orders of magnitude. This relationship is probably due to metabolic variation in quinone concentrations in bacterial cells in different environments, whereas PLFA concentrations are relatively stable under different conditions. We also found a good agreement in the community structure classifications based on the bacterial PLFAs and RQs. These results strengthen the application of both compounds as quantitative bacterial biomarkers. Moreover, the bacterial PLFA- and RQ profiles revealed a comparable dissimilarity pattern of the sampled sediments, but with a higher level of dissimilarity for the RQs. This means that the quinone method has a higher resolution for resolving differences in bacterial community composition. Combining PLFA and quinone analysis as a complementary method is a good strategy to yield higher resolving power in bacterial community structure.

## Introduction

Microbial biomass in marine sediments accounts for 0.18−3.6% of Earth’s total biomass (4.1 petagram carbon), and their community composition is highly diverse due to variation in oxygen concentrations in the overlying water, sediment carbon content, and sediment depth [1], [2]. Sediment bacteria fulfill an important role in organic matter remineralization [3], [4] and nutrient cycling [5], and are an integral component of food-webs, particularly those that are detritus-based [6], [7]. Sediment bacterial communities are more diverse than planktonic communities, and respond actively to environmental conditions of their habitat [8]–[10]. Studies on the role of bacteria in sediment biogeochemistry particularly require a quantitative assessment of both bacterial biomass and community composition.

Nevertheless, studies on estimates of bacterial biomass, community composition, and diversity are constrained by the methodological limitation that over 99% of the total bacterial population cannot be cultivated by traditional culture techniques [11]–[13]. In the past few decades, the rise of culture-independent techniques (molecular approach and chemical analysis-based method) has allowed us to further reveal sediment microbial ecology. Molecular approaches that are highly suited for high resolution description of bacteria communities in marine sediment are rDNA clone libraries, denaturing gradient gel electrophoresis (DGGE), and terminal restriction fragment length polymorphism (T-RFLP). In recent years, the advent of high-throughput sequencing technologies (e.g. pyrosequencing and Illumina) has greatly enhance the knowledge on bacterial community structure [14], [15]. As most powerful quantitative molecular approaches, the Q-PCR approach has been widely applied to quantify gene copy number as a proxy of bacterial abundance [16]–[18], and the FISH technique has been used for visualizing and quantifying bacterial cells in sediments [19], [20]. Both quantitative approaches are distinctly suitable for targeting specific phylogenetic groups but less suitable for analysis of the full bacterial community, because quantitative application for analysis of all bacterial groups requires the use of many target-specific primers and probes and also need to optimize its protocol for each target group. Moreover, the PCR-based approaches cannot eliminate methodological biases, and nucleic acid extraction from sediment samples has inherent biases, for instance extraction efficiency from sample and bacterial species [21].

Despite the superiority of molecular approaches for the analysis of bacterial community structure, also phospholipid-derived fatty acid (PLFA) analysis [22], [23] and the quinone profiling method [24], [25] have been successfully used as a chemotaxonomic analytical-based method to quantify bacterial biomass and to profile the bacterial community composition in marine sediments. PLFAs are essential membrane lipids of microbial cells and therefore proxies for bacterial biomass. Microorganisms contain numerous PLFAs with some being “general” and unspecific, while others are more specific and found in higher abundance in some microbial groups [23], [26]. Respiratory quinones (RQ, including ubiquinone [UQ] and menaquinone [MK]), and photosynthetic quinones (including phylloquinone [K1] and plastquinone [PQ]) are lipid coenzymes used for electron transfer in microbial cell membranes. A bacterial phylum has generally only one dominant molecular species of respiratory quinone (e.g. [27]). The main advantage of both chemotaxonomic methods is that there are established and standardized quantitative extraction protocols available [28], [29] that allow rapid and quantitative extraction from various types of sediment samples. Therefore, the lipid analysis is easily applicable to quantify bacterial biomass for a wide range of marine sediments without optimizing the extraction method. In fact, concentrations of bacteria-specific PLFAs have been used as a proxy for total bacterial biomass in various marine sediments (e.g. [30]), and the total RQ concentration has been found to correlate with bacterial biomass in soil [31], with the total bacterial cell count in various environments [32], and with the bacterial cell volume in lake water [33].

Moreover, analysis of PLFA- and quinone profiles has been widely utilized as a valuable tool for showing differences between samples and also community shift during experimental/monitoring periods (e.g. [34], [35]). Cluster analysis for characterizing bacterial community structure based on dissimilarity- or similarity value matrix of PLFA- and quinone profile for marine sediments showed a similar clustering pattern with that of the molecular techniques [34], [35]. One major disadvantage of both lipid analyses for studies of microbial ecology is the lower phylogenetic resolution for identifying bacterial groups than molecular approach. Thus, the lipid analysis has been combined with molecular approach as a means of overcoming the limitation of low phylogenetic resolution [24], [35], [36]. A major advantage of the PLFA technique is that it can be combined with carbon stable isotope analysis to identify active bacterial groups and to trace carbon flows in both benthic and pelagic food webs via bacteria and other microbial groups, such as microalgae, to higher trophic levels (e.g. [23]). In addition, the quinone profiling method is also possible to identify active bacterial groups by combination with carbon radioisotope labelling [37].

For marine sediments, PLFAs have been widely used as quantitative bacterial biomarkers [30], but applications of quinones as biomarkers for sedimentary bacteria are very few [24], [25]. In addition to their potential as a proxy for bacterial biomass, the ratio between quinones and PLFAs may also provide a proxy for the level of activity of the bacterial community [38], because PLFAs are structural biomass components while quinone concentrations are related to biomass and respiratory activity as they are part of electron transport chains (e.g. [39]). The ratio between total RQ and total PLFA was firstly applied in estuarine and deep sea sediments by Hedrick and White [38]. Until now, very few studies have applied this potential proxy in other aquatic systems [40], [41].

In this study we compared and evaluated PLFAs and quinones as quantitative bacterial biomarkers for bacterial biomass and community structure in marine sediments. We also explored whether the ratio between these two bacterial biomarkers could be used as a potential proxy for bacterial activity, by examining the concentrations of PLFAs and quinones and quantity and quality of organic matter in a wide range of marine sediments from the intertidal zone to the deep sea.

## Materials and Methods

### Study Areas and Sampling Procedure

Samples were collected from a wide variety of sediments, ranging from intertidal to coastal, shelf and deep-sea sediments (*see* Table 1 for the sites and sampling depth details and Table S1). Samples selected for this study came from previous published and unpublished studies were either PLFA or quinone analysis had already been performed and we completed the data set by additional analyses. No specific permissions for all sampling were required for these locations. Intertidal sediments were collected from 3 locations (Oude Bietenhaven, Zandkreek, and Rattekaai) in the Oosterschelde, a marine embayment in the SW of the Netherlands, and one location (Kapellebank) in the nearby Scheldt Estuary. Sediment was sampled manually at low tide using cores (30 cm height and 6 cm in diameter) and cores were sliced. Another tidal flat location was sampled for long-term incubations in the laboratory. In short, lab incubation sediments were collected from the surface (0∼2 cm) of a tidal flat (Biezelingse Ham) in the Scheldt Estuary, homogenized, and incubated for up to 261 days *in vitro* with regular sampling in a similar manner of the experiment that is described in [42]. North Sea sediments were collected from three stations in November 2010. Stations NS-1 and -3, located close to the Dutch coast and on the Dogger Bank respectively, are non-depositional areas, while station NS-2, situated on the Oyster Ground, is a semi-depositional area. Sediment was sampled with cores by multi-corer (Octopus type).

**Table 1:** Sample codes and characteristics.

Site	Code	Water depth (m)	Sediment depth* (cm)	*n* **	Analysis	
					OC***	DI****
**Dutch intertidal (DI):**						
Oude bietenhaven	DI-N-OB	-	0–2	2	n	n
Zandkreek	DI-N-Z	-	0–5	2	n	n
Rattekaai	DI-N-R	-	0–1.5	2	n	n
Kapellebank	DI-N-K	-	0–2	2	n	n
Lab incubations	DI-L	-	0–2	1–12	y	n
**North Sea (NS):**						
Station 1	NS-1	12	0–1	1	y	n
Station 2	NS-2	45	0–9	1–6	y	n
Station 3	NS-3	27	0–9	1–6	y	n
**Japanese coast (JC):**						
Natural	JC-N	6–83	0–1 or 0–2	1–9	y	y
Fish farm	JC-FF	30–75	0–2	1–14	y	y
**Arabian Sea (AS):**						
Station 1	AS-1	989	0–2	1	y	n
Station 2	AS-2	1700	0–2	1	y	n
**Galicia Bank**	GB	1900	0–1	1	y	n

*Total sampled depth range;

**n, sample number;

***OC: organic carbon content,

****DI: degradation index. Additional information is shown in Table S1.

Japanese natural coastal sediments were collected from nine different bays and embayments in the Seto Inland Sea using a Smith-McIntyre Grab sampler or an Ekman-Berge grab sampler, and then subsampled by collecting surface sediment samples from late September to early October 2008 and from early May to early June 2009. Japanese fish farm sediments were collected from 14 stations located in and around fish farming areas in the north part of Sukumo Bay, located in Sikoku, Japan in the same manner as the coastal sediments collected from the Seto Inland Sea.

Deep sea sediments were collected from the Arabian Sea and the Atlantic Ocean. Samples from the Arabian Sea were obtained from two stations, with one station (AS-1) being situated within the oxygen minimum zone (OMZ) (i.e. <9 µM O_2_ in the overlying water) and the other station (AS-2) below the OMZ (i.e. oxic bottom water) in January 2010 [43]. Sediment from the Atlantic Ocean was sampled at the Galicia Bank off the coast of Spain in September–October 2008 [44].

All sediment samples were either directly stored frozen (−20°C) or freeze-dried and subsequently stored frozen (−20°C) until extraction and analysis of PLFAs, quinones, and organic carbon.

### Chemotaxonomic Markers of PLFAs and RQs in Different Groups of Bacteria

Important chemotaxonomic PLFA and RQ markers for bacteria are listed in Table 2. In this study, we defined the sum of saturated fatty acid (SFAs, C_13_–C_18_), branched fatty acids (BFAs), and mono-unsaturated fatty acids (MUFAs, ≤C_19_) as total bacterial PLFA. In addition, there are various other bacteria-*specific* PLFAs, for instance i17∶1ω7 is for a marker for the genus *Desulfovibrio* [45], but these compounds are typically present in low concentrations, which precluded analysis of these compounds in most sample sets in the present study.

**Table 2:** Major fingerprints of PLFA and quinone as a marker for different bacterial groups in this study.

Biomarker	Proteobacteria					Bacteroidetes	Actinobacteria
	Alpha-	Beta-	Gamma-	Delta-	Epsilon-		
**PLFA** a							
SFA (C_12_–C_19_)^c^	G	G	G	G	G	G	G
i14∶0							
i15∶0				++M		+++	++
i16∶0						+	+++
i17∶0				++M		+	+
a15∶0				+M		+M	+
a17∶0				+M			++
10Me16∶0				++M			+M
10Me17∶0							+M
10Me18∶0							++M
cy17∶0	+^d^	+	+	+++M		+	
cy19∶0	+		+	+M			
16∶1ω7c	G	G	G	G	G	G	G
18∶1ω9c	G	G	G	G	G	G	G
18∶1ω7c	+++	+	+++	++M	+++		
**Ref. no.**	[67]–[69]	[70]–[72]	[73], [74]	[45], [75]	[76], [77]	[70], [78], [79]	[80]
**Quinone** b							
UQ-8	++++*	++++M	++++M				
UQ-9	++++*		++++M				
UQ-10	++++M						
MK-*n* (*n*≤8)		++++*	++++*	++++M	++++M (MK-6)	++++M	++++*
MK-*n* (*n*≥9)						++++*	++++M
MK-*n*(H*_x_*)				++++*			++++M
**Ref. no.**	[67]–[69]	[72], [81]	[74], [82], [83]	[84], [85]	[86]	[70], [79], [87]	[80], [88], [89]

In this study, we refer to the different quinones with the following abbreviations: ubiquinone - UQ-*n*; and menaquinone - MK-*n*. The number (*n*) indicates that of the isoprene unit in the side chain of the quinone. Partially hydrogenated MKs were expressed as MK-*n*(H*_x_*), where *x* indicates the number of hydrogen atoms saturating the side chain.

a PLFA data were modified mainly from [23], [26], [90], [91].

b Quinone data were modified mainly from [56], [92]–[94].

c Saturated fatty acids.

d +, 1–5%; ++, 5–15%; +++, 15–40%; ++++, >40% of total PLFA pool or total quinone pool; *, present in few species; G, a maker found in a broad range of bacteria and algae, and M, a marker can be used specifically as an indicator for specific bacterial group with the phylum.

### PLFA Extraction and Analysis

PLFAs were extracted from freeze-dried sediment (∼4 g) and analyzed as described in [28]. In short, total lipids were extracted from the sample in chloroform–methanol–water (1∶2∶0.8, v/v) using a modified Bligh and Dyer method and fractionated on silicic acid into different polarity classes. The methanol fraction, containing phospholipids, was derivatized using mild alkaline methanolysis to yield fatty acid methyl esters (FAMEs), which were recovered by hexane extraction. FAME concentrations were determined by gas-chromatography-combustion-isotope ratio mass spectrometry (GC-c-IRMS) for all samples except for Japanese samples that were analysed by gas chromatography-flame ionization detection (GC-FID). The concentrations obtained by both methods are comparable from our previous experience (*r^2^* = 0.99, unpubishied data). Identification of individual FAME was based on comparison of retention times with known reference standards.

### Quinone Extraction and Analysis

Quinones were extracted from freeze-dried or frozen sediment (∼6 g) as described previously [25], [29]. The types and concentrations of each quinone were determined using a HPLC equipped with an ODS column (Eclipse Plus C18, 3.0 (I.D.) ×150 mm, pore size 3.5 µm, Agilent technologies) and a photodiode array detector (SPD-M20A, Shimadzu: for the Japanese samples, and Waters 996 for the samples from the Dutch intertidal zone, North Sea, and the deep sea). A mixture of 18% isopropyl ether in methanol was used as the mobile phase at a flow rate of 0.5 mL min^−1^. The quinone molecular species were identified by the linear relationship between the logarithm of the retention times of quinones and the number of their isoprene units, using the identification-supporting sheet, which is available upon request from T. K, based on the equivalent number of isoprene units (ENIU) of quinone components as described by [46]. Details on the analytical conditions have been described by [33].

### Organic Carbon Content

For determination of the organic carbon (OC) content, sediment samples were first freeze-dried or dried at 60°C in an oven overnight, acidified to remove carbonate, and further vacuum-dried. The OC content of the sediment was determined with an elemental analyzer (NA-1500n, Fisons, Rodano-Milan, Italy: for the Japanese sediments and FlashEA 1112, Thermo Electron, Bremen, Germany: for the sediments of other samples).

### HAA Extractions, Analysis and Calculation of Degradation Index

For the Japanese sediments, concentrations of hydrolysable amino acids (HAAs) were analyzed as described in [47] and used to calculate the degradation index (DI), a proxy for the quality, or “freshness”, of the organic matter in the sediment. Briefly, samples (∼1 g) of freeze-dried sediment were washed with 2 M HCl and Milli-Q water and then hydrolyzed in 6 M HCl at 110°C for 24 h. After neutralization by 1 M NaOH, amino acids were derivatized with *o*-phthaldialdehyde (OPA) [48] prior to injection to reverse-phase high-pressure liquid chromatography (HPLC). Amino acid concentrations were measured by HPLC and further details on the analytical conditions have been described by [47]. The DI was calculated following [47]:
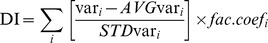
where var*_i_*, AVGvar*_i_*, STDvar*_i_*, and fac.coef*_i_* are the mol%, mean, standard deviation and factor coefficient of amino acid *i*, respectively. The factor coefficient was described in [49].

### Cluster Analysis of the Pattern of Differences Among Samples in Individual PLFAs and RQs

We conducted a cluster analysis to identify groups of similar bacterial PLFA and RQ patterns. We first normalized the mole fraction of bacterial PLFA and RQ (Z*_j,i_*), because this analysis depends on the absolute values of the data, using the following normalization equations [50]:
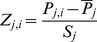



With:
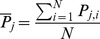


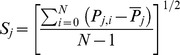
where *P_j,i_* is the mole fraction of bacterial PLFA or RQ component *j* and sample *i*, *N* is the number of samples, and 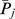 and *S_j_* are the average value and the standard deviation of the mole fraction of bacterial PLFA or RQ among samples, respectively. After normalization, the average value 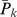 and the standard deviation *S_k_* are shown respectively as 0 and 1, where *k* is the normalized component of bacterial PLFA or RQ. We used both>1 mol% of component to the bacterial PLFA (without general bacterial compounds (SFAs (C_12_−C_19_), 16∶1ω7c, and 18∶1ω9c), MUFAs (≥C_20_), and PUFAs) or RQ profile, >30% of coefficient of variance of compound among all samples for this data analysis, and reconstructed profiles, because general and minor components interfere with the result of this analysis. As results, the cluster analysis was conducted based on the mole fraction of 12 bacterial PLFAs and 16 RQ molecular species among all samples (*see* “Cluster analyses of bacterial PLFA and RQ profiles”). The normalized values were used to produce a cluster dendrogram based on the Euclidean distance matrix, and the dendrogram was constructed using Ward’s method with the graphing program KyPlot version 5.0 (KyensLab Inc., Tokyo, Japan).

### Cluster Analysis Based on the Full Profiles of the Bacterial PLFAs and RQ

We conducted another cluster analysis to compare sample discrimination and its resolution based on bacterial PLFA or RQ profiles. A dissimilarity index (*D*) of profile was calculated using the following equation [51].
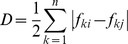
where *n* is the number of PLFA or RQ component. In the PLFA profiles, *f_ki_* and *f_kj_* are the mole fractions of the *k* PLFA component for the *i* and *j* samples, respectively. In RQ profiles, *f_ki_* and *f_kj_* are the mole fractions of the *k* RQ component for the *i* and *j* samples, respectively (*f_ki_*, *f_kj_*>1 mol%; Σ*f_ki_* = Σ*f_kj_* = 100 mol%). Cluster analysis was performed with the program KyPlot version 5.0 based on the *D* distance matrix and a dendrogram was constructed using the between-groups linkage method. Values≤0.1 of *D* of RQs are not recognized as different RQ profiles according to the analytical precision based on the duplicate analytical results including extraction and measurement process (97% statistical reliability) [52]. For the PLFA analysis, we determined the threshold value, 0.13, in the same manner as the value of the quinone profiling method (*see* [52]) using 12 duplicate results of the incubation sediment samples (Fig. S1).

### Statistical Analysis

Spearman’s rank correlations (*r_s_*) were used to show the relationships among bacterial PLFA concentration, RQ concentration and organic carbon content and the relationships between OC content and DI. Pearson’s correlation coefficients (*r*) were used to show the relationships between OC content and RQ/bacterial PLFA ratio and between DI and RQ/bacterial PLFA ratio. Analysis with Spearman’s rank correlation and Pearson’s correlation coefficient was performed using the statistical program PASW Statistics for Windows version 18J (IBM Japan, Tokyo, Japan). Mantel tests were used to test the significance of the correlation between dissimilarity matrices based on bacterial PLFA or RQ profiles, using the R package [53].

## Results

### PLFA and Quinone Concentrations

**Figure 1:**
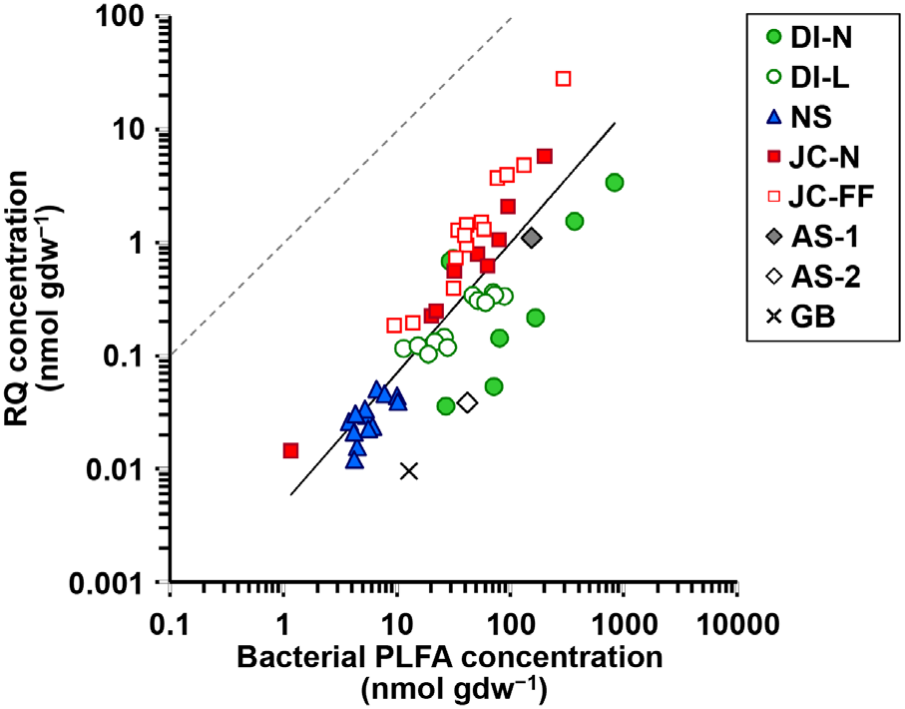
Comparison between bacterial PLFA and RQ concentration in the sediment with different sample sets. Line indicates trend for the full dataset. The dotted line indicates the 1∶1 relationship.

Total bacterial PLFA concentrations (i.e. the sum of SFAs [C_13_–C_18_], BFAs, and MUFAs [≤C_19_]) in the sediment varied over three orders of magnitude (range 1.2–834 nmol gdw^−1^) with lowest values for Japanese natural coastal sediment (JC-N-9) and highest values for Dutch intertidal natural sediment (Fig. 1 and Table 3). Total RQ concentrations in the sediment ranged from 0.01 to 28 nmol gdw^−1^ with lowest values for the Galicia bank (GB) and highest values for Japanese fish farm sediment, and were one to two orders of magnitude lower than the bacterial PLFA concentrations (Fig. 1 and Table 3). RQ concentration showed a positive log-log correlation with the bacterial PLFA concentration for the full dataset as well as within the individual sample sets (Fig. 1 and Table 4). However, there were clear differences in slopes of the fits for the individual sample sets with the highest slope for the Japanese fish farm sediments (1.487) (i.e. relatively rich in RQs) and the lowest slope for the Dutch intertidal incubation sediments (0.716) (i.e. relatively rich in PLFAs) (Table 4). Two deep sea samples from the Arabian Sea (AS-2) and GB were relatively far from the overall trend line with relatively high PLFA concentrations and low RQ concentrations (Fig. 1).

**Table 3:** Concentration of bacterial PLFAs, respiratory quinones (RQ) and organic carbon (OC) in marine sediments in this study.

	Bacterial PLFA (nmol gdw^−1^)		RQ (nmol gdw^−1^)		OC (mg-C gdw^−1^)	
	Range	Mean ± SD	Range	Mean ± SD	Range	Mean ± SD
**All samples**	1.17−834	67.0±122	0.01−28.0	1.22±3.75	0.37−60.4	8.8±11.2
**Dutch intertidal (DI):**						
Natural	27.2−834	202±280	0.03−3.4	0.84±1.14	-*	-
Lab incubations	11.5−89.3	43.0±26.2	0.10−0.36	0.23±0.11	3.6−16.3	9.8±6.2
**North Sea (NS):**	3.84−10.3	6.05±2.2	0.01−0.05	0.03±0.01	0.44−3.0	1.6±1.2
**Japanese coast (JC):**						
Natural coast	1.17−202	63.3±30.2	0.01−5.8	1.26±1.8	0.37−23.7	10.1±8.6
Fish farm	9.55−295	68.8±72.9	0.18−28.0	3.54±7.19	1.7−49.6	10.5±11.7

*Not determined.

**Table 4:** Log/log power regressions and Spearman’s rank coefficients between the bacterial PLFA (nmol gdw^−1^) and respiratory quinone (RQ) concentrations (nmol gdw^−1^), and between organic carbon (mg-C gdw^−1^) and the bacterial PLFA and quinone concentration of individual sample set.

	Bacterial PLFA (*x*)		OC (*x*)		OC (*x*)	
	*versus* RQ (y)		*versus* bacterial PLFA (y)		*versus* RQ (y)	
	Power regression	*r_s_*	Power regression	*r_s_*	Power regression	*r_s_*
**All samples**	*y = *0.0049×^1.151^	0.823**	*y = *6.326×^0.882^	0.946**	*y = *0.039×^1.148^	0.809**
**Dutch intertidal (DI):**						
Natural	*y = *0.0096×^0.774^	0.643	−	−	−	−
Lab incubations	*y = *0.0156×^0.716^	0.825**	*y = *5.991×^0.861^	0.781**	*y = *0.045×^0.720^	0.982**
**North Sea (NS):**	*y = *0.0059×^0.899^	0.624*	*y = *5.650×^0.138^	0.253	*y = *0.028×^0.115^	0.263
**Japanese coast (JC):**						
Natural coast	*y = *0.0094×^1.122^	0.983**	*y = *6.027×^1.011^	1.000**	*y = *0.069×^1.149^	0.983**
Fish farm	*y = *0.0043×^1.487^	0.952**	*y = *5.962×^1.022^	0.880**	*y = *0.056×^1.570^	0.847**

Levels of significance are *P<0.05, **P<0.01.

### Relationship Between Organic Carbon Contents and Bacterial PLFAs and RQs

**Figure 2:**
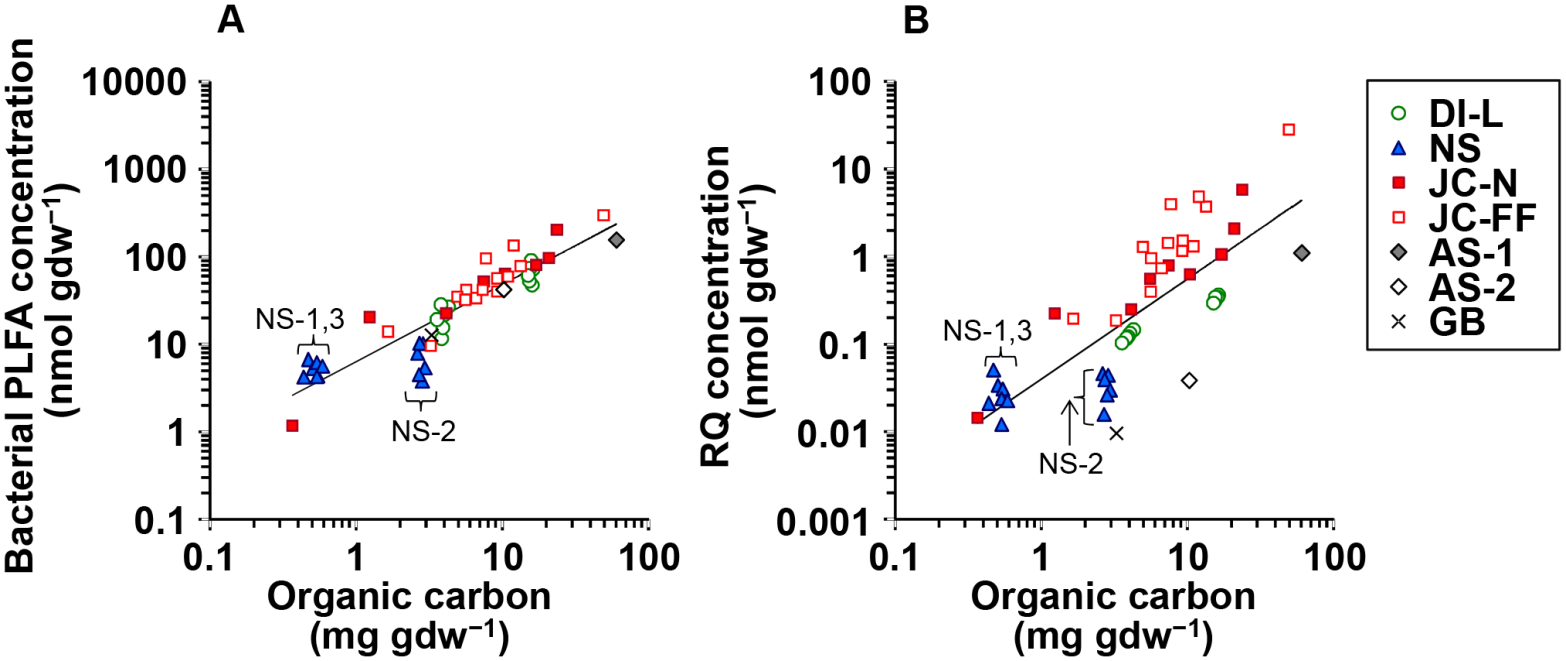
Comparisons between: a) organic carbon and bacterial PLFA concentration, b) organic carbon and RQ concentration.

The sediment organic carbon (OC) content ranged from 0.4 to 60 mg gdw^−1^ (mean 8.8±11, mg gdw^−1^ *n* = 51) over more than two orders of magnitude in all samples (Table 3). A positive power correlation between the OC contents and the bacterial PLFA concentrations was observed (Fig. 2a and Table 4). This correlation was similar for all sample sets, except for the Dutch intertidal incubation and North Sea samples (Table 4). Given the correlation, it was not surprising to find that the correlation between the OC contents and the RQ concentrations was also positive (Fig. 2b and Table 4).

### RQ/PLFA Ratios

**Figure 3:**
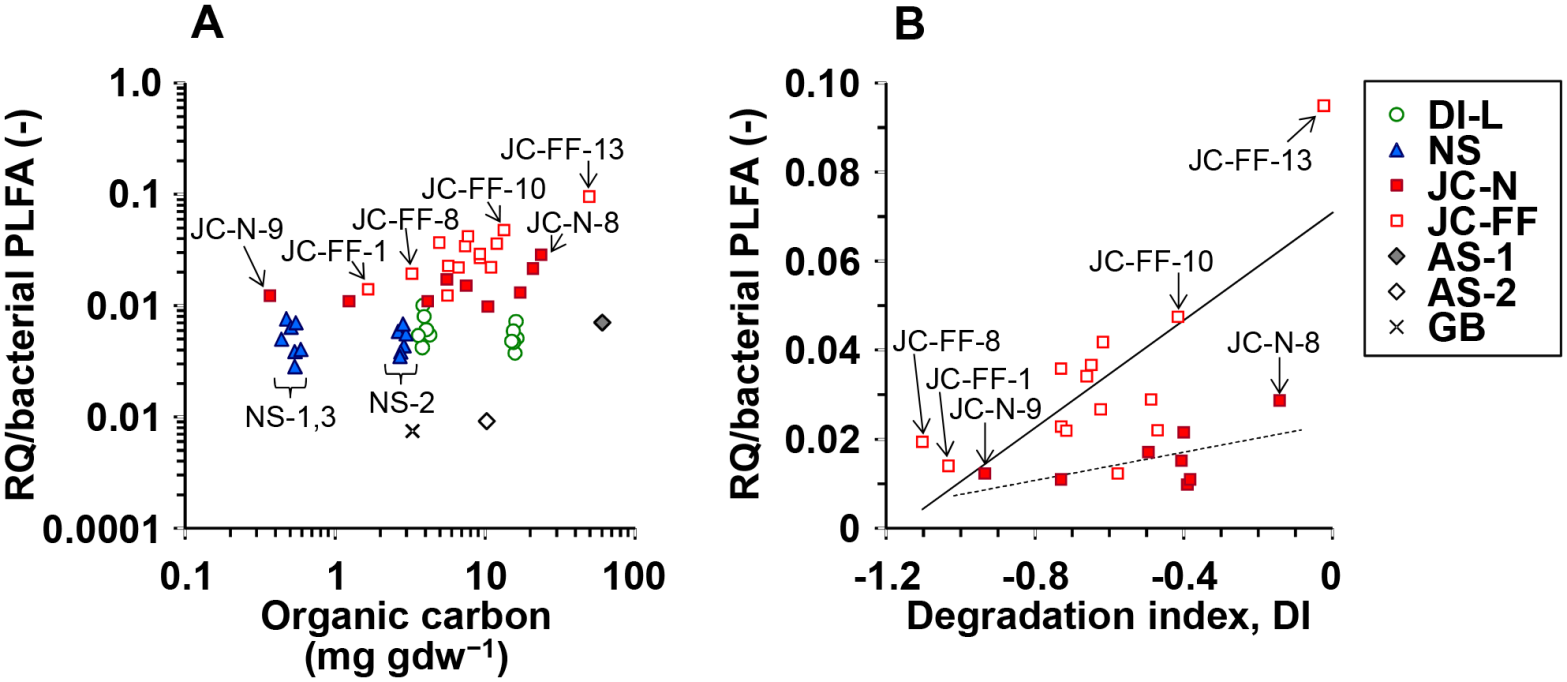
Relationships between: a) organic carbon and RQ/bacterial PLFA ratio in the sediment with different sample sets, b) degradation index and RQ/bacterial PLFA ratio in the Japanese coastal natural- and fish farm sediments.

We used a ratio based on mole concentration between total RQ and total bacterial PLFA (RQ/bacterial PLFA). The ratios of RQ/bacterial PLFA ranged from 0.0007 to 0.095 with lowest value for the deep sea sediment (GB) and highest values for Japanese fish farm (JC-FF-13) (Fig. 3a). Strong positive log-log correlations between the OC contents and the RQ/bacterial PLFA ratios of the Japanese fish farm samples were observed (*r* = 0.888, *P*<0.01) (Fig. 3a), while ratios for the other sample sets, except deep sea samples, showed no significant correlation with OC content.

### Degradation Index Values for Japanese Sediments

DI values for all Japanese samples were ranged strongly from −1.1 to −0.2 (Fig. 3b) with more negative values indicating more degraded (refractory) material. The DI value was positively correlated with the OC content (*r_s_* = 0.738, *P*<0.05 for Japanese natural coast and *r_s_* = 0.702, *P*<0.01 for Japanese fish farm), meaning the OC in the sediments with the highest OC content was relatively fresh (labile). A positive linear correlation between DI and the RQ/bacterial PLFA ratios only for the Japanese fish farm sediments was observed (*r* = 0.751, *P*<0.01) (Fig. 3b), whereas there was no significant correlation for Japanese natural coastal sediments (*r* = 0.665, *P* = 0.072). Note that the positive relationship for Japanese fish farm sediments was due to the sample from Stn. JC-FF-13 (without the plot of Stn. JC-FF-13, *r* = 0.469, *P* = 0.106).

### Relative Composition of PLFA and Quinone Pools

**Figure 4:**
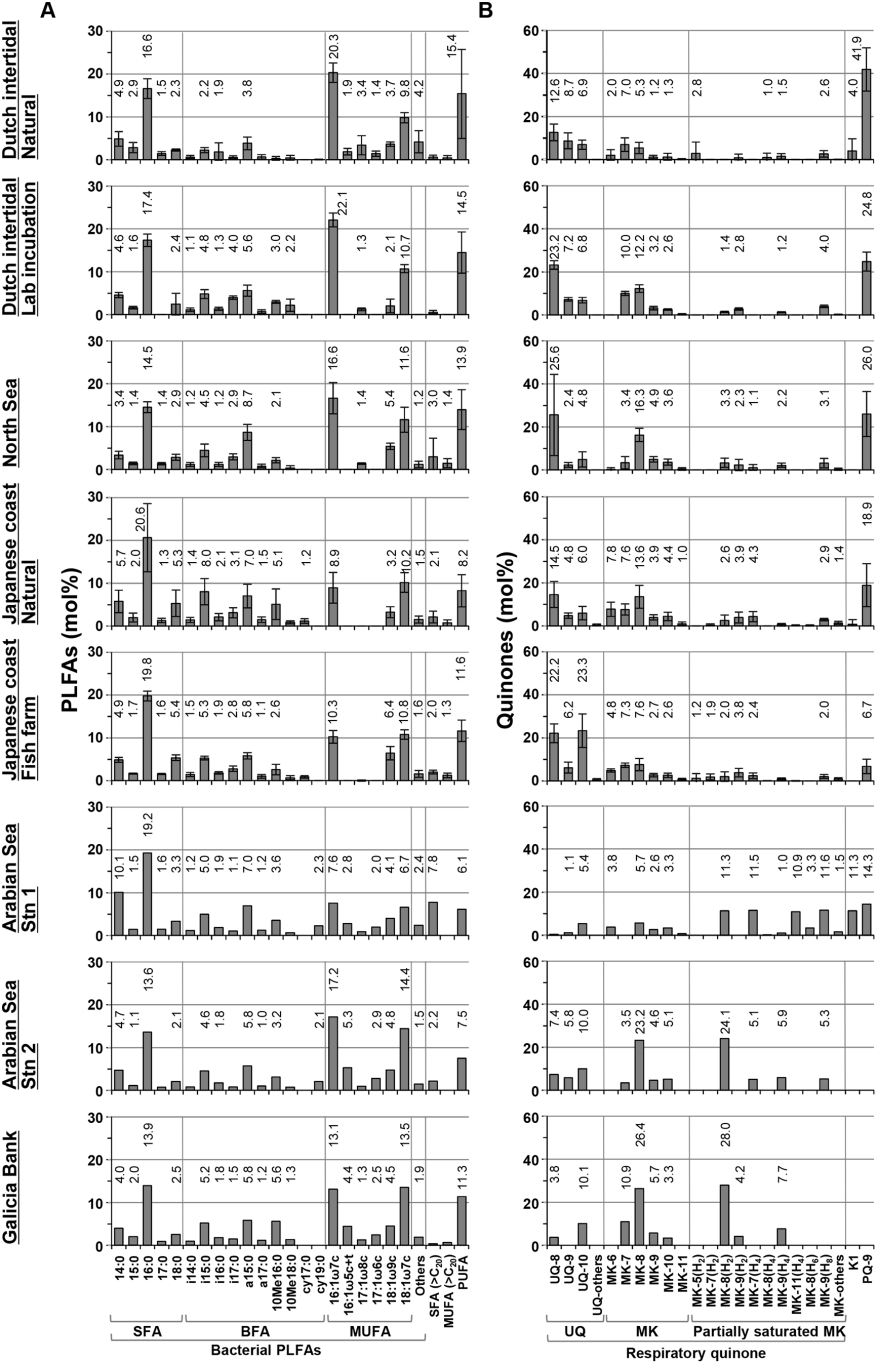
Summarized compositions of a) PLFAs and b) quinones with different sample sets. More than 3 mol% of components to total pool of each PLFAs and RQs were indicated as others. Note that the full range of PLFAs and quinones analyzed is shown here, meaning that this includes both bacteria-specific and non-specific compounds.

The composition of the bacterial PLFA (general [SFAs (≤ C_19_), 16∶1ω7c, and 18∶1ω9c] + specific) in the sediment showed less variation as compared to the composition of RQ (Fig. 4). The three dominant PLFAs, 16∶0, 16∶1ω7c and 18∶1ω7c, were present generally in almost all the samples (Fig. 4a). SFAs (≤ C_19_), 16∶1ω7c, and 18∶1ω9c as a *general* marker for bacteria accounted for 57 mol% of the total bacterial PLFA pool in all samples (range 47–71 mol%). Bacteria-*specific* PLFAs showed variation in the full dataset. Together, i15∶0, a15∶0, 10Me16∶0, and 18∶1ω7c as a *specific* marker for bacterial groups accounted for average 25 mol% (range 11–46 mol%) of the total bacterial PLFA pool in all samples (Fig. 4a).

In general, the relative composition of RQ varied more strongly (Fig. 4b). The most obvious difference is seen between the deep-sea and the other (coastal and estuarine) sediments. Almost all coastal sediments except Japanese fish farm sediments were dominated by PQ-9 and UQ-8, while two deep sea sediments (AS-2 and GB) were dominated by MK-8(H_2_) and MK-8. Japanese fish farm sediments were dominated by UQ-10 and UQ-8. Together, UQ-8, -9, and -10 accounted for 45 mol% (range 9–83 mol%) of the total RQ pool in all samples. PQ-9 and K1, which are derived from photosynthetic organisms, were observed in not only coastal area, but also in the oxygen minimum deep-sea sediment (AS-1) (Fig. 4b).

### Cluster Analysis of the Pattern of Differences Among Samples in Individual PLFAs and RQs

**Figure 5:**
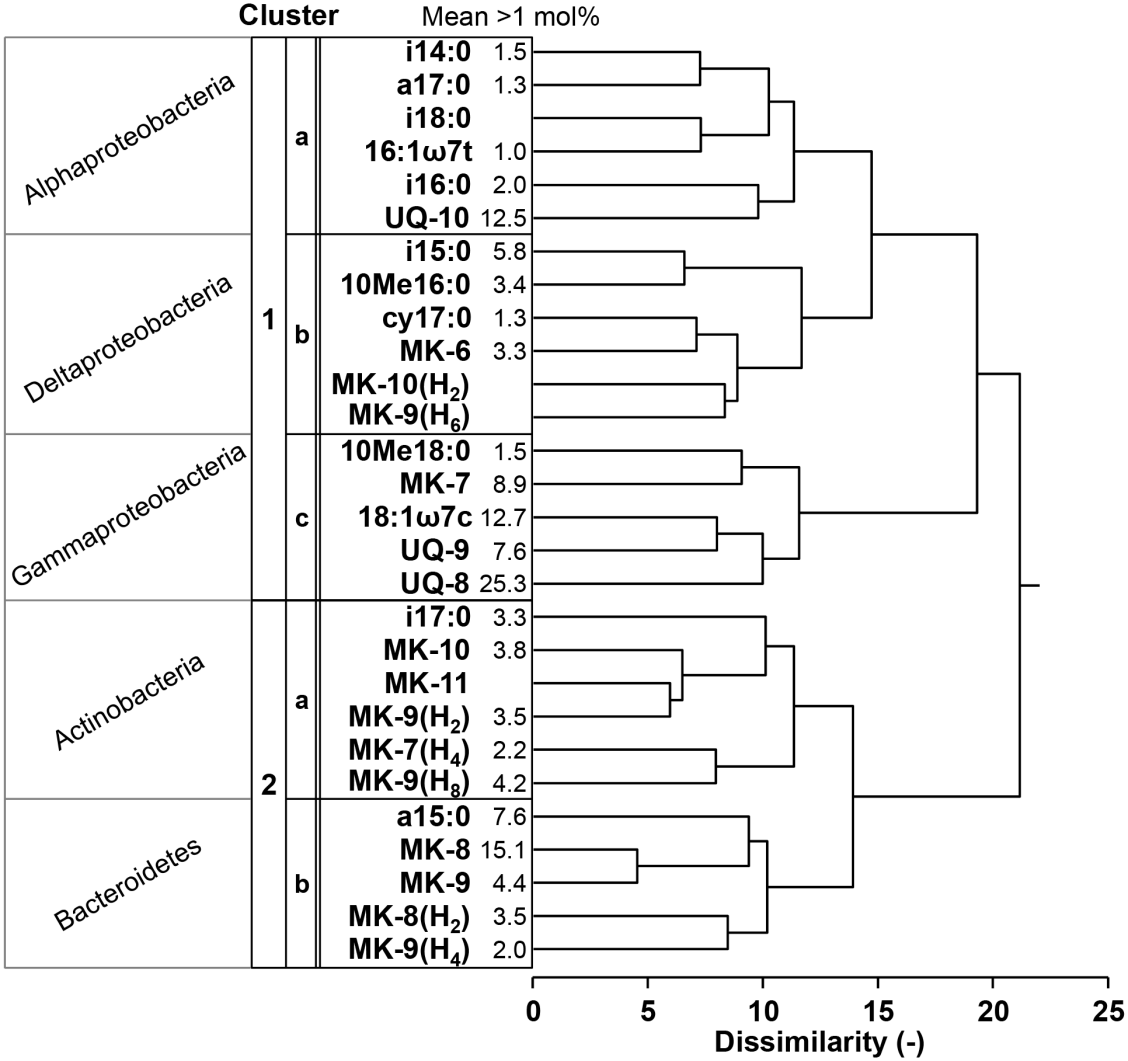
Cluster analysis of the pattern of differences among samples in the individual bacterial PLFAs and RQs. The mean mole percentage value indicates the mean mole fraction among all samples.

The differences in the bacterial PLFA and RQ profiles for the different sample sets (Fig. 4) were further clarified by two cluster analyses. The first analysis was performed to investigate the co-variation in the relative abundance of the individual bacteria-*specific* PLFAs (sum of BFAs and MUFAs (≤C_19_) except 16∶1ω7c and 18∶1ω9c) and RQs (Fig. 5). When different compounds cluster closely, this indicates that these compounds are probably derived from the same bacterial groups. Two main clusters (cluster-1 and -2) were observed that were further divided into two sub-clusters (cluster-1a, -1b, 2a, and -2b) (Fig. 5). These five clusters were characterized by a relatively high mole fraction of group-specific bacterial PLFAs and RQs among all samples. It is noteworthy that UQs were present in cluster-1, whereas almost all partially saturated MKs were in cluster-2.

### Cluster Analyses of Bacterial PLFA and RQ Profiles

**Figure 6:**
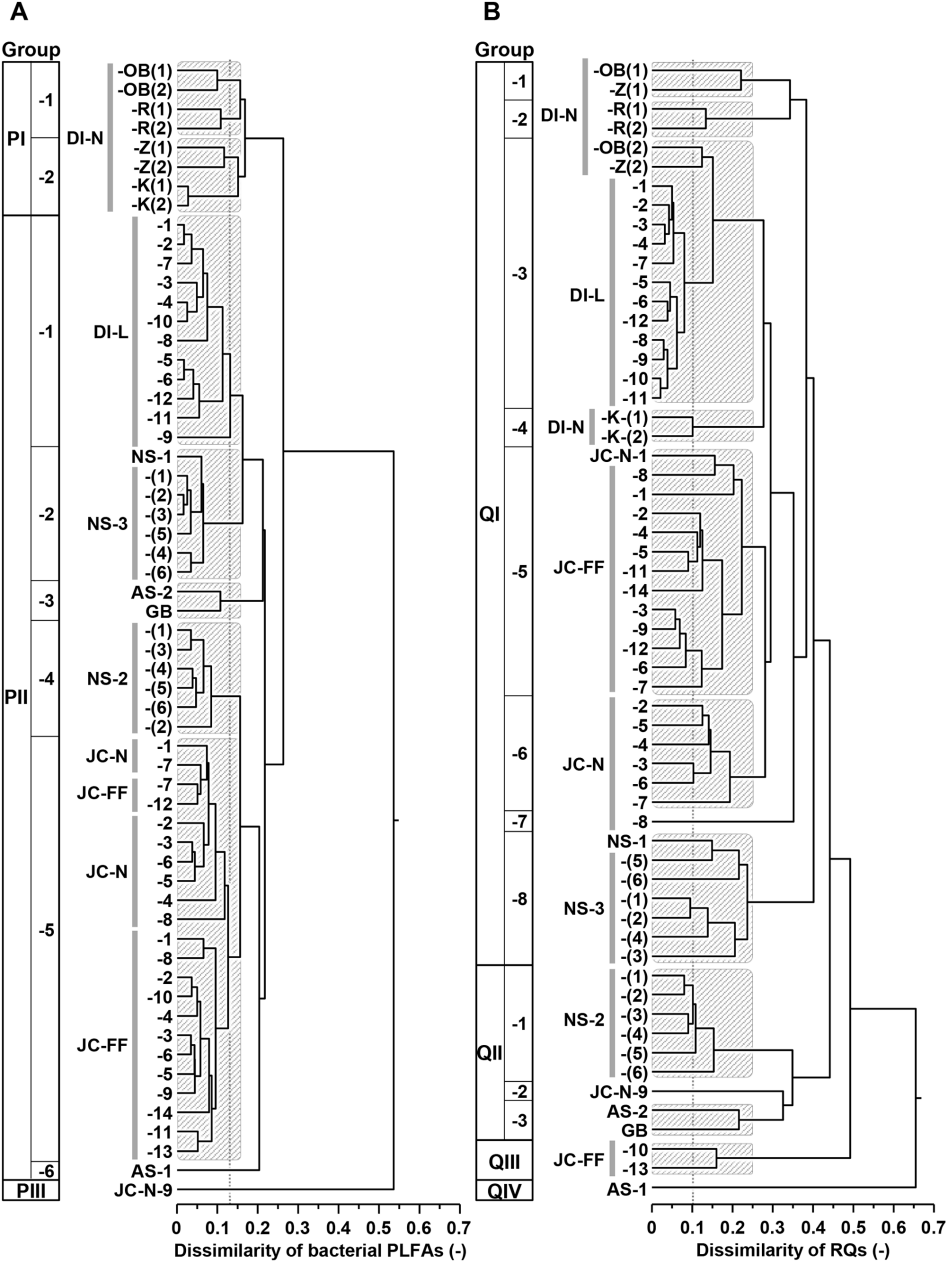
Classification of profiles based on the dissimilarity value matrix data calculated from the mole fractions of a) the bacterial PLFAs and b) the RQs of the sediments. Abbreviation of each sample indicates the system and other information of the sample (*see* Table 1). Parentheses in the abbreviation indicate the depth layer at the sampling station.

The second cluster analysis was conducted to investigate the differences in bacterial community structure of the different sediments based on the bacterial PLFA and RQ profiles separately in order to compare the chemotaxonomic resolution of these two methods (Fig. 6). The bacterial PLFA profiles clearly separated into three main groups (group PI, PII, and PIII in Fig. 6a). Group PI comprised all Dutch intertidal natural sediments, while all other samples were included in group PII. The only exception here is a single Japanese natural coastal sediment sample (JC-N-9) that formed a separate cluster (PIII). Further differentiation involved division of group PII into six different groups (group PI-1∼6 in Fig. 6a) based on the threshold value of 0.13 (representing the observed level of dissimilarity between replicate samples, *see* Fig. S1). The RQ profiles were divided clearly into four main groups (group QI, QII, QIII, and QIV in Fig. 6b). Within these main groups, almost all sample sets were distinguished as separate groups based on the threshold value of 0.1 for sample discrimination of different RQ profiles [52] (Fig. 6b). The general sample classification of the different sediments between bacterial PLFAs versus RQs based on the dissimilarity index was significantly correlated (using 10,000 randomizations, Mantel’s coefficient *r* = 0.435, *P* = 0.0001).

## Discussion

Analysis of lipid biomarkers is a powerful tool for quantification of bacterial abundance and community structure. While PLFAs have been widely utilized as quantitative bacterial biomarkers in marine sediment [22], [23], applications of the quinone profiling method to marine sediments are still very few [24], [25]. In this study, we analyzed concentrations of PLFAs and RQs in a broad range of marine sediments to investigate and compare their application as indicators of bacterial biomass and community composition.

### PLFAs and RQs as Bacterial Biomass Indicators

We found a strong correlation between total concentrations of the bacterial biomarkers PLFAs and RQs across several orders of magnitude, both for the individual sample sets and for the whole dataset (Fig. 1). PLFA concentrations have been used frequently as a measure of bacterial biomass in seawater and marine sediments (e.g. [22], [23], [54]), because PLFA concentrations are relatively constant in bacterial biomass and PLFAs degrade rapidly upon death of the source organism, meaning that they are specific for *living* bacterial biomass [23]. The strong correlation across several orders of magnitude between PLFAs and RQs indicates that RQs also provide an estimate of *living* bacterial biomass in sediment. This allowed us to determine the conversion from RQ concentration (nmol gdw^−1^) to bacterial biomass (mg C gdw^−1^; biomass = 0.192 RQ^0.586^, *r_s_* = 0.853, *P*<0.001, *n* = 59). The equation is based on the correlation between the RQ concentration and summed concentrations of four bacteria-specific PLFAs (i14∶0, i15∶0, a15∶0 and i16∶0) calculated by the equation detailed in [30] using the conversion factors from [55]. Interestingly, this relationship is not linear, which is probably due to metabolic variation in quinone concentrations in bacterial cells in different environments.

Previous studies have already demonstrated that total RQ concentrations correlated very well with microbial biomass carbon in soil (measured by a fumigation-extraction method, *r* = 0.96, [31]), with the total bacterial cell count in various environments (*r* = 0.98, [32]), and with bacterial cell volume in lake water (*r* = 0.98, [33]). Our study is the first to demonstrate the good correlation between concentrations of RQ versus PLFAs as a compound-specific biomarker for bacteria in marine sediment. Thus, these results indicate that RQ concentration can be utilized as a proxy for bacterial biomass in sediment samples.

Despite the overall strong correlation between PLFAs and RQs, a more detailed look at Fig. 1 reveals that there is residual variation to be explained. Firstly, the range in RQ concentrations was around one order of magnitude higher than that of the bacterial PLFA concentrations in the full sample set. Secondly, the slopes of the fits for the individual sample sets were different (Fig. 1 and Table 4), which implies that the different sediments contained bacterial communities with different RQ/PLFA ratios. We consider two possible explanations for this varying RQ/PLFA ratio. The first explanation is inherent group-specific differences in the RQ/PLFA ratios of the different groups of bacteria contributing to the overall bacterial community. This can be related, for example, to the type of energetic metabolism of the bacteria (e.g. [56]). The second explanation concerns the activity of the bacteria. While bacterial PLFA concentrations (being a structural biomass component) are relatively stable under different conditions [57], [58], concentrations of RQs in bacterial cells can also depend on the metabolic activity due to growth phase [59], substrate utilization [60], and redox state [61]. The strong PLFA versus RQ correlation over a broad range of sediments suggests that RQ concentration reflects mainly bacterial biomass but that may have an additional component related to the activity/metabolism of bacteria.

If RQ concentrations are also dependent on the activity of the bacteria, RQ concentrations relative to PLFA concentrations (the RQ/bacterial PLFA ratio) may also depend on the quality and quantity of the OM in the sediment as these two factors directly influence bacterial activity [36], [62], [63]. We investigated this relationship through assessment of the correlation between the RQ/bacterial PLFA ratio versus OM *quantity* (total OC content) for all samples and OM *quality* (i.e. DI, the amino acid-based degradation index) for the Japanese sediments (Fig. 3b). The absence of a clear correlation between OC content and the RQ/bacterial PLFA ratio for the full dataset (Fig. 3a) indicates that this ratio was not influenced by OM *quantity*. In addition, we also investigated the relationship between the RQ/bacterial PLFA ratio versus OM *quality* for the Japanese samples. The OM *quality* was determined by the degradation index (DI), which is based on the relative composition of hydrolysable amino acids in the sediment [47]. This index provides an indication of the quality (or ‘freshness’) of the organic matter in the sediment with most negative values indicating relatively low quality (or ‘refractory’) material. Despite the wide range of observed DI values (−1.10 to −0.02), which indicates substantial variation in OM *quality* between samples for both the natural and fish farm sediments (Fig. 3b), there was no correlation between DI values and the RQ/bacterial PLFA ratio for the natural sediments and only a weak correlation for the fish farm sediments (Fig. 3b). Overall, our results indicate that there was no strong control of bacterial activity on the RQ/PLFA ratio by both quantity and reactivity of the OC pool.

Still, the RQ/bacterial PLFA ratios for Japanese fish farm sediments were clearly higher than the natural sediments. According to previous studies, the ratio between total RQ and total PLFA concentration has been used to indicate mainly two aspects: a presence of aerobic bacteria and facultative heterotrophic bacteria and a respiratory activity in comparison with fermentation processes [38], [40], [41]. Further investigation of the RQ/PLFA ratio, combined with a study on bacterial metabolism in marine sediments, is needed to explain the observed residual variation and the role of these two aspects.

### Linking PLFA and Quinone Biomarkers

The cluster analysis as shown in Fig. 5 was conducted to investigate the co-variation between the bacterial PLFAs and RQs in the different sediments and their association with specific bacterial groups. The cluster analysis showed two main groups: cluster-1 comprised UQs, which are specific for Gram-negative Proteobacteria (*see* Table 2). Cluster-2 comprised almost all partially saturated MKs, which are predominantly present in Gram-positive bacteria, thereby indicating that cluster-2 was dominated by Gram-positive bacteria (Fig. 5 and Table 2). Based on the taxonomic assignment of PLFAs and RQs in Table 2, the subcluster can be analyzed in more detail. Subcluster-1a comprised UQ-10, indicating that this cluster was dominated by members of the class Alphaproteobacteria. Subcluster-1b was characterized by the presence of MK-6, i15∶0, 10Me-16∶0, and cy17∶0, indicating that this cluster was predominance of the class Deltaproteobacteria. Subcluster-1c was characterized by UQ-8 and 18∶1ω7c, indicating that it comprised mainly members of the class Gamma- and Beta-proteobacteria. Betaproteobacteria are well known to be a minor group in marine sediment [36], therefore, Subcluster-1c must have been dominated by mainly members of the class Gammaproteobacteria. Cluster-2a was characterized by MK-10, MK-9(H_8_), and i17∶0, indicating that this cluster is relatively rich in members of the Actinobacteria. Subcluster-2b was characterized by MK-8, MK-9, and a15∶0, indicating that this cluster comprised members of the Bacteroidetes. Our study is the first to demonstrate a general agreement in the chemotaxonomic classification based on bacterial PLFAs versus RQs. This strengthens the use of these biomarkers for characterization of the sediment bacterial community. Although taxonomic resolution of both analyses is limited to identify phylogenetic groups of bacteria (low phylogenetic resolution), the value of this approach can be in combination with stable isotope probing to allow researchers to trace the flow of elements within communities [23].

### Bacterial Communities of the Different Sediments

The cluster analyses as shown in Fig. 6 were performed to investigate the resolution of the two types of bacterial biomarkers and their ability to distinguish between bacterial communities from different sediments. In general, the bacterial PLFA- and RQ profiles revealed a similar classification pattern in bacterial community differences in our wide range of marine sediments (Fig. 6). However, there is a clear difference in the resolution of both methods with sample classification based on the RQ profile distinguishing 37 groups, whereas classification based on of the bacterial PLFA profile distinguishes only 13 groups. In other words, the level of dissimilarity between RQ profiles was substantially higher than the level of dissimilarity between the PLFA profiles.

The higher sample discrimination in the RQ profile can be explained by the higher specificity of the RQs for specific bacterial groups (*see* Table 2) as well as the more pronounced differences between RQ profiles of different bacterial groups (that are typically dominated by one RQ while PLFA profiles typically comprised 5∼17 PLFAs) [38], [64], [65]. In addition to this general trend, there were also some notable differences in the classification of the deep sea sediments (AS-1, AS-2, and GB), Japanese natural coastal sediment (JC-N-9), Japanese fish farm sediment (JC-FF-10 and JC-FF-13), North Sea sediment (NS-2), and Dutch natural intertidal sediment (DI-N-K) (Fig. 6).

Two groups of RQs that were particularly important for the higher level of dissimilarity in RQ profiles are UQ-*n* and the partially saturated MKs. UQs are good markers for Alpha-, Beta-, and Gamma-proteobacteria, whereas as for PLFAs, the only specific marker for proteobacteria is 18∶1ω7c (*see* Table 2). Partially saturated MKs, which exist in Actinobacteria, Deltaproteobacteria, and Epsilonproteobacteria, showed most variation between sample sets, especially, MK-8(H_2_), MK-9(H_2_), MK-7(H_4_), MK-9(H_4_) and MK-9(H_8_) which were present in more than 29 samples (15.4±9.2 mol% in the total RQ pool). Actinobacteria generally show a larger variation in MKs than PLFAs (e.g. [66]). Thus, higher sample discrimination in RQ profile could be due to presence of compounds originated from members of mainly Proteobacteria (UQ-*n*) and Actinobacteria (MK-*n*(H*x*)).

Overall, we demonstrated that both concentration of bacterial PLFAs and RQs are good indicators for bacterial biomass and RQ profile can discriminate community difference more clearly than the bacterial PLFA profile. Thus, the combination between PLFA analysis (in combination with the stable isotope probing (SIP) technique) and quinone profiling method is a good strategy for studies on the role of bacteria in sediment biogeochemistry. These methods, and their applications, can be further expanded with the development of a method for stable isotope analysis of quinones so that quinones can also be applied in combination with stable isotope probing.

## Supporting Information

Figure S1 **Analytical precision of total bacterial PLFA pools using dissimilarity values from the two PLFA pools resulted from duplicate analyses.**
(TIF)

Table S1 Sample codes and characteristics.
(DOCX)
