# Supplementary figures and images for: Phospholipid-Derived Fatty Acids and Quinones as Markers for Bacterial Biomass and Community Structure in Marine Sediments
Source: PLoS One. 2014 Apr 25;9(4):e96219. doi: 10.1371/journal.pone.0096219 (PMC4000199; doi:10.1371/journal.pone.0096219)

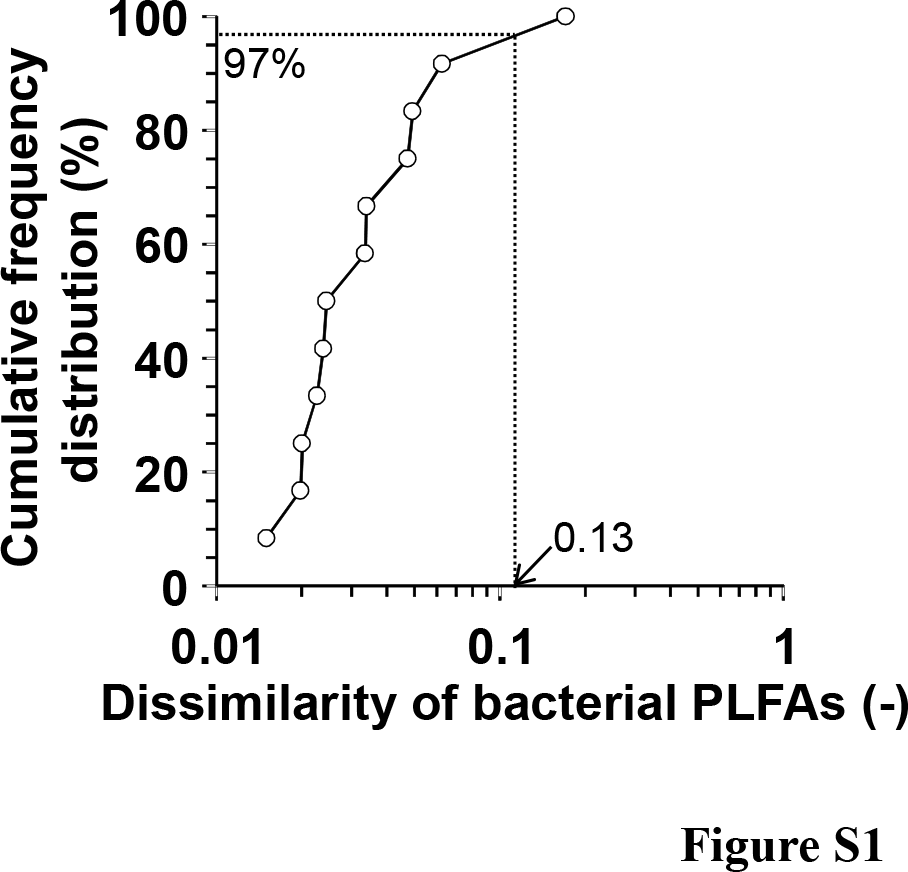

Supplement: Figure S1 — Analytical precision of total bacterial PLFA pools using dissimilarity values from the two PLFA pools resulted from duplicate analyses. (TIF) [file pone.0096219.s001.tif]
