# Supplementary material for: Phospholipid-Derived Fatty Acids and Quinones as Markers for Bacterial Biomass and Community Structure in Marine Sediments
Source: PLoS One. 2014 Apr 25;9(4):e96219. doi: 10.1371/journal.pone.0096219 (PMC4000199; doi:10.1371/journal.pone.0096219)
Supplement: Table S1 — Sample codes and characteristics. (DOCX) [file pone.0096219.s002.docx]

Table S1. Sample codes and characteristics.

| **Site** | **Code** | **Latitude** | **Longitude** | **Comments** |
| --- | --- | --- | --- | --- |
| **Dutch intertidal (DI):** |  |  |  |  |
| Oude bietenhaven | DI-N-OB | 51°26.87'N | 4°5.79'E | Two depth layers |
| Zandkreek | DI-N-Z | 51°32.68'N | 3°53.37'E | Two depth layers |
| Rattekaai | DI-N-R | 51°26.35'N | 4°10.18'E | Two depth layers |
| Kapellebank | DI-N-K | 51°27.52'N | 3°58.81'E | Two depth layers |
| Lab incubations | DI-L | - | - | Incubation over 261 days |
| **North Sea (NS):** |  |  |  |  |
| Station 1 | NS-1 | 55°10.03'N | 3°9.04'E |  |
| Station 2 | NS-2 | 54°8.09'N | 4°20.05'E | Six depth layers |
| Station 3 | NS-3 | 53°24.09'N | 5°9.0'E | Six depth layers |
| **Japanese coast (JC):** |  |  |  |  |
| Natural | JC-N | 33°7.7'~34°36'N | 131°4.8'~135°18'E | Nine bays and embayments |
| Fish farm | JC-FF | 32°55.1~56.8'N | 132°30.3~35.6'E | Fish-farming area |
| **Arabian Sea (AS):** |  |  |  |  |
| Station 1 | AS-1 | 22°32.9'E | 64°2.4'E | Oxygen minimum zone |
| Station 2 | AS-2 | 22°18.5'E | 63°24.5'E |  |
| **Galicia Bank** | GB | 42°27.64'E | 10°39.28'W |  |
